# Emerging roles for the autophagy machinery in extracellular vesicle biogenesis and secretion

**DOI:** 10.1096/fba.2020-00138

**Published:** 2021-03-02

**Authors:** Andrew M. Leidal, Jayanta Debnath

**Affiliations:** ^1^ Department of Pathology and Helen Diller Family Comprehensive Cancer Center University of California San Francisco San Francisco CA USA

## Abstract

Autophagy classically functions to maintain cell health during stressful conditions by targeting cytosolic components for degradation and recycling via lysosomal pathways. However, accumulating evidence also supports roles for autophagy‐related genes (ATGs) in non‐degradative processes including cellular secretion. Here, we review emerging roles for the autophagy machinery in regulating extracellular vesicle loading and secretion and discuss how functional coupling of these pathways may impact normal physiology and disease.

## 1 INTRODUCTION

Extracellular vesicles (EVs) are a heterogenous population of membrane‐bound nano‐sized structures released from cells into the extracellular environment. Originally described as a mechanism to selectively eliminate proteins, nucleic acids, and lipids from cells, EVs are now viewed as vehicles of intercellular communication that can impact diverse physiological and pathological processes.^1^, ^2^, ^3^, ^4^ The cell autonomous and non‐autonomous effects of EVs are inherently linked to the biomolecules that are packaged and released within these structures. As a result, EV cargoes have been profiled extensively in many biological contexts, owing largely to their potential clinical utility as biomarkers for liquid biopsy.^5^, ^6^ Nevertheless, the precise cellular mechanisms that specify diverse biomolecules for secretion via EVs and the regulatory pathways that direct the EV secretion and biogenesis machinery remain poorly understood.

Emerging evidence indicates that autophagy, a highly conserved process of cellular self‐digestion essential for homeostasis and adaptation to stress, may direct cargo secretion via certain extracellular vesicle (EV) sub‐populations.^7^, ^8^ In adverse conditions, autophagy facilitates cell survival via clearance of damaged molecules and mobilization of intracellular stores of energy and nutrients. However, recent discoveries have revealed that autophagy pathway components also regulate processes that are related, but functionally distinct from canonical autophagy.^8^, ^9^ Indeed, accumulating evidence implicates autophagy in protein secretion via the classical secretory pathway and so‐called unconventional pathways that bypass the endoplasmic reticulum (ER) and Golgi apparatus.^10^ Here, we focus on emerging functions of the autophagy machinery in unconventional secretion via EVs and discuss potential roles for this pathway in normal physiology and disease.

## 2 OVERVIEW OF THE CLASSICAL AUTOPHAGY PATHWAY

Autophagy classically refers to a collection of related cellular self‐digestion processes that target the cytoplasmic material for breakdown in the lysosome.^11^, ^12^ The three main routes of autophagy include macroautophagy, microautophagy, and chaperone‐mediated autophagy (CMA), which are largely defined by how cytosolic material is delivered to lysosomes. Macroautophagy involves the sequestration of cargo within double‐membrane vesicles called autophagosomes, which subsequently fuse with lysosomes to mediate cargo degradation.^11^ Microautophagy, the least‐studied form of autophagy, occurs through direct invagination and engulfment of cytoplasmic material at the limiting membrane of endosomes or lysosomes.^13^ Finally, CMA, a form of autophagy only found in mammals, proceeds through chaperone complexes that deliver cytosolic targets to the limiting membrane of lysosomes, where they are unfolded and translocated into the lumen by lysosome‐associated receptor protein type 2A (LAMP2A) to facilitate breakdown.^13^ This review largely focuses on the molecular machinery required for macroautophagy, hereafter termed autophagy, and its emerging role in specifying cargo for secretion in EVs.

**FIGURE 1:**
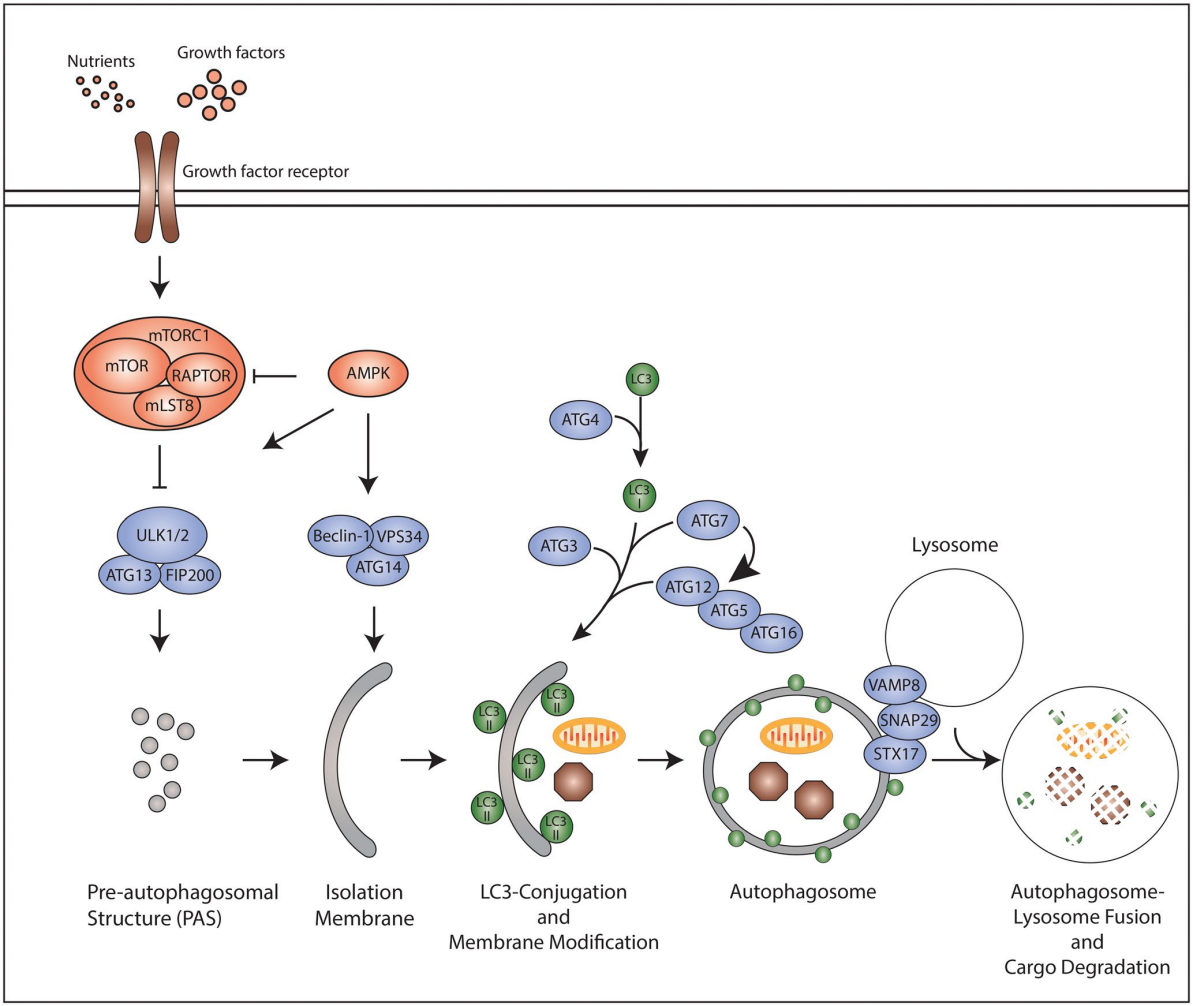
The classical autophagy pathway. Signals are integrated by the core autophagy machinery, which hierarchically regulate individual steps within the autophagy pathway. Formation of the pre‐autophagosomal structure (PAS), the first step in the autophagy pathway, is controlled by the ULK1‐ATG13‐FIP200 kinase complex. Nutrients and growth factors (e.g. amino acids, IGF‐1) trigger signaling through growth factor receptors. Ultimately, these signals converge upon the mammalian target of rapamycin complex 1 (mTORC1) comprised of mTOR, RAPTOR and mLST8, as well as AMPK, which reciprocally modify the ULK complex to regulate its functions in PAS formation. The PAS is subsequently modified by the Beclin‐1‐ATG14‐VPS34 complex to mediate formation of the isolation membrane. Expansion of the isolation membrane is associated with two ubiquitin‐like reactions involving ATG7, ATG5, ATG12, ATG16 and ATG3 which ultimately conjugate phosphatidylethanolamine (PE) to microtubule‐associated protein 1 light chain 3 (MAP1LC3B; also known as LC3) and other ATG8 family proteins. LC3‐PE targets LC3 to autophagosomal membranes where it facilitates membrane expansion and cargo sequestration. Finally, the autophagosome double‐membrane is sealed and captured cargo targeted to the lysosome through autophagosome‐lysosome fusion. In this schematic, arrows indicate activating signals, whereas blunt‐end lines represent inhibitory signals

The molecular machinery that orchestrates autophagy is encoded by AuTophaGy‐related genes (ATGs) evolutionarily conserved from yeast to mammals.^14^ ATG proteins assemble into functional complexes that are sequentially recruited to control the individual steps of autophagosome formation and maturation (Figure 1). Autophagy is regulated by diverse signaling networks, most notably mTORC1 and AMPK, which coordinately inhibit “self‐eating” when conditions are optimal and activate the pathway in response to stresses such as nutrient starvation.^15^ These signals are integrated by an autophagy initiation complex comprised of the ULK1, FIP200, and ATG13, which upon activation is recruited to the pre‐autophagosomal assembly site (PAS) and serves to stimulate the local production of phosphatidyl‐inositol 3‐phosphate and membrane nucleation via the BECN1‐ATG14L‐VPS34 class III PI3‐kinase complex.^12^ The expansion of nascent autophagosomal membranes is associated with two ubiquitin‐like conjugation pathways that require ATG3, ATG5, ATG7, ATG12, and ATG16 and ultimately conjugate phosphatidylethanolamine (PE) to ATG8 family proteins including microtubule‐associated protein 1 light chain 3B (MAP1LC3B; also known as LC3) (Figure 1). This process, commonly termed LC3‐conjugation, targets PE‐conjugated LC3 (also called LC3‐II) to autophagosomal membranes where it is required for membrane expansion and recruitment of cargo, most notably proteins that contain a short‐sequence motif termed an LC3‐interaction region (LIR).^12^ Once sealed, double‐membrane autophagosomes are trafficked along microtubules by dynein–dynactin motor complexes to lysosomes.^16^ The final step of the autophagy pathway is achieved by membrane fusion between the autophagosome and lysosome and requires soluble N‐ethyl maleimide sensitive factor attachment protein receptors (SNAREs) including STX17, VAMP8, and SNAP29^17^ (Figure 1). Cargo delivered to the lysosome is then broken down and recycled to the cytoplasm.

## 3 AUTOPHAGY‐RELATED PATHWAYS THAT MEDIATE CARGO DEGRADATION

In addition, ATG proteins regulate degradative pathways that are related to, but clearly distinct from, classical autophagy. Recent discoveries have revealed that the autophagy machinery can also target LC3 to single‐membrane vesicles including endosomes, lysosomes, phagosomes, macropinosomes, and entotic vacuoles to facilitate degradation of intracellular and engulfed extracellular material.^18^, ^19^, ^20^, ^21^ LC3 is targeted to these vesicular organelles through mechanisms that are independent of the ULK1‐FIP200‐ATG13 complex and autophagosome formation.^20^, ^22^, ^23^ Instead, these pathways rely on a subset of ATGs including the ubiquitin‐like LC3‐conjugation machinery for the delivery of LC3 to single‐membranes with ATG16L playing a critical role in targeting autophagy components to atypical membrane sources.^24^

**FIGURE 2:**
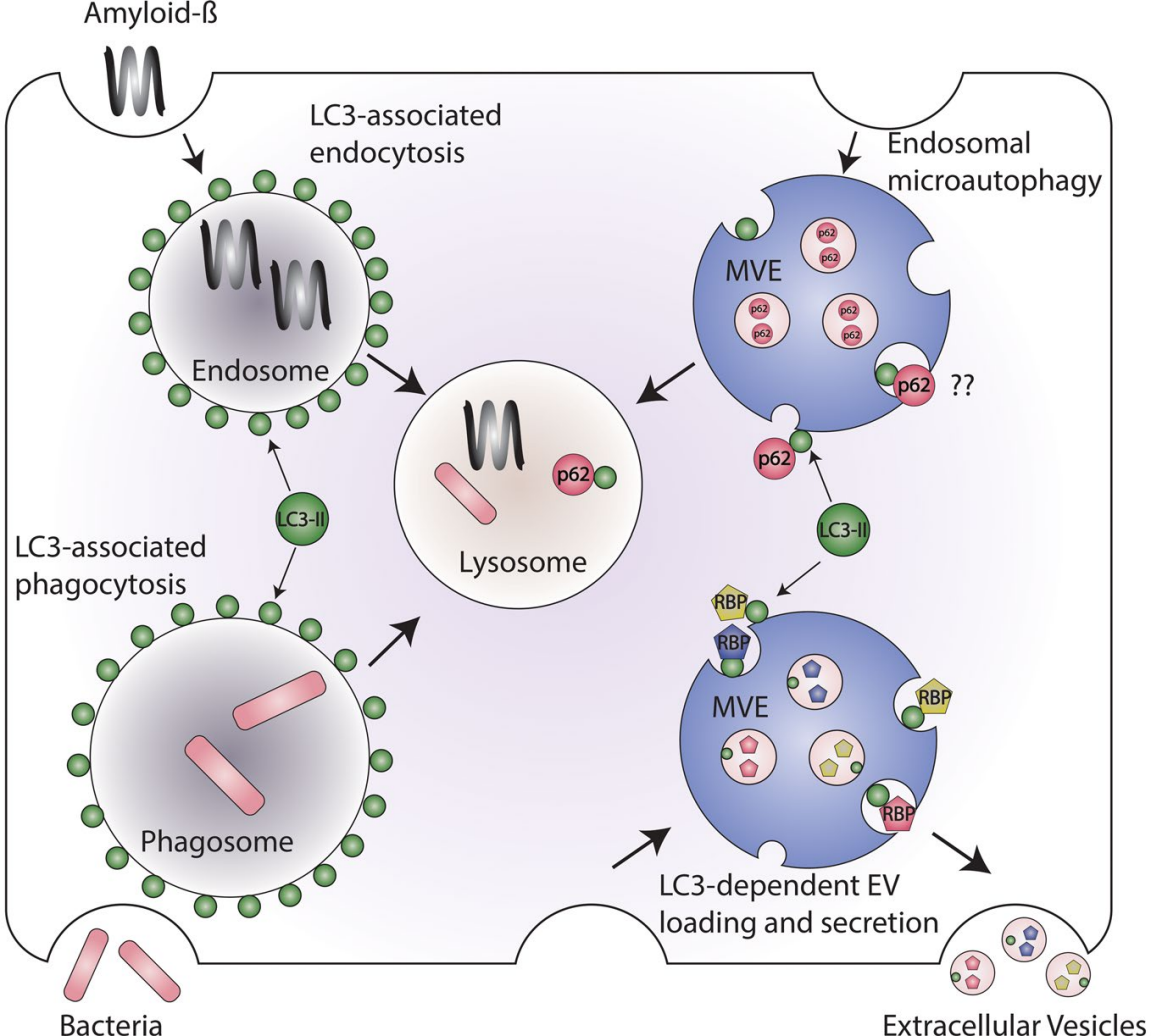
Emerging autophagy‐related pathways that employ LC3‐conjugation to single‐membrane endocytic and phagocytic vesicles. Components of the autophagy conjugation machinery, in addition to regulating classical autophagy, facilitate the delivery of microtubule‐associated protein 1 light chain 3 (MAP1LC3B; also known as LC3) and other ATG8 family proteins to single‐membrane vesicles including phagosomes, endosomes, and multivesicular endosomes (MVEs). During LC3‐associated phagocytosis (LAP) and LC3‐associated endocytosis (LANDO), LC3 delivery to the surface of phagosomes and endosomes, respectively, controls the trafficking and lysosomal degradation of material engulfed from the extracellular space such as bacteria or amyloid‐β. In contrast, LC3 is targeted to discrete subdomains at the limiting membrane of MVEs during endosomal microautophagy (eMI) and LC3‐dependent EV loading and secretion (LDELS), which undergo budding to form small intraluminal vesicles. LC3 facilitates the packaging of RNA‐binding proteins (RBPs) into these intraluminal vesicles, which subsequently are released as extracellular vesicles in the LDELS pathway. LC3 may also mediate the packaging of autophagy cargo receptors such as p62/SQSTM1 into intraluminal vesicles during endosomal microautophagy, but it remains unclear whether such pathways contribute to EV secretion

The autophagy machinery operates at single‐membrane organelles to direct protein degradation through at least three mechanistically distinct processes: LC3‐associated phagocytosis (LAP), LC3‐associated endocytosis (LANDO), and endosomal microautophagy (eMI) (Figure 2). Functional studies have revealed that LAP is critical in phagocytic cells for the clearance of pathogens and damage‐associated molecules, whereas LANDO facilitates the degradation of extracellular proteins such as amyloid‐β (Aβ) taken up via receptor mediated endocytosis.^25^, ^26^ eMI is triggered by external stresses including nutrient starvation and osmotic shock and is implicated in the turnover of cytosolic components and integral membrane proteins such as TRPML1^18^, ^27^ (Figure 2). Although LAP, LANDO, and eMI share a common requirement for the LC3 conjugation machinery, the function and fate of LC3 within these pathways dramatically differ. During LAP and LANDO, LC3 is conjugated to nearly the entire cytosolic surface of phagosomes and endosomes, respectively, to regulate the trafficking and lysosomal degradation of engulfed extracellular material^23^, ^26^ (Figure 2). The separation of LC3 and extracellular cargo on opposing sides of endosome and phagosome membranes prevents LC3 from being degraded during LAP and LANDO and suggest that the autophagy machinery primarily regulates vesicular trafficking within these two pathways. In contrast, LC3 is delivered to subdomains at the limiting membrane of multivesicular endosomes (MVEs) during eMI^18^, ^27^ (Figure 2). These subdomains subsequently undergo intraluminal budding via the endosomal sorting complexes required for transport (ESCRT) machinery, delivering LC3, and interacting cargo into the cisternae of MVEs in the form of intraluminal vesicles (ILVs), which are then broken down upon MVE‐lysosome fusion. Notably, LC3 and related family members appear critical for eMI^18^, ^27^ and likely coordinate the recruitment and sorting of cargo, such as p62/SQSTM1 and TRPML1, into ILVs to mediate their degradation in the endolysosomal compartment (Figure 2).

## 4 SECRETORY AUTOPHAGY

Although the autophagy machinery is predominantly proposed to target proteins for lysosomal degradation, increasing evidence implicates ATGs in diverse pathways mediating cellular secretion, which have been collectively termed “secretory autophagy.” Indeed, genetic loss‐of‐function studies support roles for ATGs in the conventional secretion of cytokines and membrane transporters, but the precise mechanism underlying these phenotypes has remained obscure.^28^, ^29^, ^30^ Furthermore, the autophagy machinery has been implicated in the unconventional secretion of proteins lacking amino‐terminal signal sequences.^10^ While most eukaryotic secretory proteins traffic through the endoplasmic reticulum (ER) and Golgi apparatus for extracellular secretion, there is an expanding group of proteins released through unconventional mechanisms that bypass the ER and/or Golgi. The link between autophagy and unconventional secretion was first observed in yeast, where a subset of ATGs is required for the extracellular release of acyl‐coA‐binding protein Acb1 (AcbA in Dictyostelium discoideum).^31^, ^32^ Subsequent studies have extended this functional link to mammalian systems and revealed a diverse array of targets that are released via ATG‐dependent unconventional secretion (secretory autophagy) including the pro‐inflammatory molecules IL‐1β and IL‐18 and high mobility group protein B1 (HMGB1),^33^ lysozyme,^34^ cathepsins,^35^ insulin‐degrading enzyme (IDE),^36^ and the iron carrier protein ferritin.^37^

Evidence from unconventional secretion paradigms implicates autophagosome precursor membranes in trafficking and extracellular release of non‐classical cargoes. In yeast, genetic dissection of Acb1 secretion has revealed requirements for LC3 and core ATGs involved with autophagosome formation including ATG1 and ATG6 (the yeast orthologues of ULK1 and BECN1), whereas ATGs necessary for autophagosome‐vacuole fusion were largely dispensable to this pathway.^31^, ^32^ Secretion of the atypical cytokine IL‐1β in mammalian cells also requires ATGs critical for early steps in the autophagy pathway.^33^, ^38^ Interestingly, recent studies have revealed that IL‐1β is targeted for secretion via translocation from the cytosol into the lumen of the endoplasmic reticulum–Golgi intermediate compartment (ERGIC), a site of LC3 conjugation and donor membrane for growing autophagosomes.^39^ This translocation process requires TMED10, a transmembrane protein proposed to multimerize into a channel that delivers IL‐1β across the ERGIC membrane. Accordingly, conditional deletion of TMED10 within murine myeloid cell lineages leads to impaired IL‐1β secretion in experimental models of sepsis.^39^ Studies have also implicated Sec22b, a regulator of ER‐Golgi protein trafficking, and the exocytic soluble NSF‐attachment protein receptors (SNAREs), SNAP23 and SNAP29, with their binding partners STX3 and STX4 in this secretory pathway.^37^ Together, these observations support a model where IL‐1β is targeted for unconventional secretion via loading into vesicular precursors of autophagosomes, which then undergo SNARE‐dependent fusion with the plasma membrane to deliver this inflammatory molecule outside the cell.

Although ATGs are genetically required for the unconventional secretion of select proteins, it is unclear how this machinery contributes to protein secretion. Most targets of secretory autophagy such as Acb1 and IL‐1β have not been identified as binding partners of LC3 or other ATGs. These observations broach that the autophagy machinery does not directly specify proteins for unconventional secretion but rather facilitates processes related to the biogenesis, trafficking, and fusion of vesicles that carry secretory cargo. Indeed, LC3 conjugation to vesicles at nascent autophagic structures is critical for membrane trafficking and fusion events that mediate autophagosome formation and maturation and may also contribute to the release of non‐classical secretory cargo at the plasma membrane and/or endosomal compartments.^40^, ^41^ At the same time, multiple LC3‐interacting proteins and well‐established targets of autophagy including p62/SQSTM1 have been identified in proteomic analyses of conditioned media and plasma.^42^, ^43^ Nevertheless, it remains unclear how these proteins are released and if these pathways require ATGs. Clearly, it will be important to determine whether targets of secretory autophagy are released through mechanisms similar to IL‐1β or via multiple, distinct ATG‐dependent pathways that function to deliver specific proteins outside the cell.

## 5 EMERGING CONNECTIONS BETWEEN AUTOPHAGY AND EV SECRETION

Studies examining the role of ATGs in unconventional secretion have uncovered novel functions of the autophagy machinery in the biogenesis, cargo loading, and secretion of EVs. Early evidence of this relationship was observed in endothelial cells, which release small EVs containing LC3, ATG16L1, and LAMP2 in response to serum starvation.^44^, ^45^ Although serum starvation is a potent inducer of endothelial cell apoptosis, small EVs bearing autophagic markers were clearly distinct from apoptotic bodies and required caspase‐3 for extracellular release. Autophagy components are also directed for EV secretion upon inhibition of the phosphoinositide kinase PIKfyve.^46^ The treatment of cells with the PIKfyve inhibitor apilimod promotes the release of LC3 and related family members and autophagy cargo receptors including p62/SQSTM1, NBR1, and OPTN. Furthermore, p62/SQSTM and the EV marker protein CD63 localize within the same MVE‐like structures within drug treated cells.^46^ Nevertheless, it remains unclear whether the autophagy machinery is functionally required for these EV secretion pathways and whether a specific autophagic vesicular intermediate contributes to the release of autophagic cargo via EVs.

More recently, core components the autophagy machinery have been genetically implicated in EV biogenesis. The non‐canonical conjugation of ATG12 and ATG3 yields a complex that interacts with the ESCRT accessory protein ALIX and regulates endosome function.^47^ Genetic disruption of ATG12–ATG3 conjugation results in altered trafficking of late endosomes, impaired EV biogenesis, and budding of virus‐like particles. ATG5 has also been shown to facilitate EV production via dissociation of the V_1 _V_0_‐ATPase complex at the limiting membrane of MVEs and late endosomes, which inhibits endosomal acidification and directs these organelles for secretion.^48^ Interestingly, ATG5 appears to regulate V_1 _V_0_‐ATPase complex dissociation by partitioning the ATP6V1E1 subunit into ILVs bud from the limiting membrane of MVEs, facilitating the release of this subunit in EVs. Together, these studies highlighted ATGs as important effectors of EV secretion pathways that are distinct from their traditional roles in classical autophagy.

## 6 LC3‐DEPENDENT EXTRACELLULAR VESICLE LOADING AND SECRETION (LDELS)

In line with this view, our group recently uncovered a novel role for LC3 and the autophagy conjugation machinery in specifying the proteins and RNAs that are loaded into EVs for secretion outside the cell.^49^ Remarkably, the lipid conjugated form of LC3 and related family members are highly enriched within a subset of EVs and required for the loading of RNA‐binding proteins (RBPs) and small non‐coding RNAs through a process termed LC3‐dependent EV loading and secretion (LDELS). This EV‐sorting pathway also requires multiple core components of the LC3‐conjugation machinery but appears independent of ATGs required for other steps in canonical autophagy, such as FIP200 and ATG14^49^ (Figure 2). In addition, LDELS functionally requires neutral sphingomyelinase 2 (nSMase2)‐dependent production of ceramide. Overall, these data support the model that LDELS is mediated by a pool of LC3 conjugated to the limiting membrane of MVEs in a process analogous to LAP, LANDO, and eMI (Figure 2). LC3 at the limiting membrane of the MVE captures cytoplasmic cargoes, such as RBPs and small non‐coding RNAs, and thereafter undergoes intraluminal budding. Subsequently, these LC3‐positive ILVs are released as EVs when MVEs fuse with the cell surface.

LC3 and related ATG8 family members appear to serve at least two important functions in the LDELS pathway. First, LC3 recruits factor‐associated with nSMase activation (FAN) through a conserved LIR, which facilitates intraluminal budding and ILV formation by stimulating nSMase2 production of ceramide^49^ (Figure 2). Whereas many EV biogenesis pathways rely upon the ESCRT machinery for membrane budding,^3^ LDELS appears to utilize an alternative mechanism in which localized ceramide production at MVEs drives inward bending of the membrane; this mechanism may only require CHMP4B for the final scission step of ILV formation. Second, LC3 is required for sorting cargo such as RBPs into EVs for extracellular release.^49^ LC3 recruits RBPs such as scaffold‐attachment factor B1 to MVEs via LIR‐dependent interactions, and LC3‐RBP complexes are packaged into EVs during the budding process (Figure 2). In support of this model, the LDELS pathway was also found to control EV secretion of small non‐coding RNAs including small nucleolar RNAs (snoRNAs) and microRNAs (miRNAs).^49^ Although the physiological relevance of LDELS and RBP secretion remain unknown, it is tempting to speculate that this pathway may serve as a cellular disposal mechanism similar to autophagy. Importantly, this cell autonomous role for LDELS does not preclude functions of this pathway in intercellular communication since cellular material incorporated into LC3‐positive EVs may trigger signaling or contain information that can impact cell fate decisions and tissue microenvironments through non‐cell autonomous mechanisms.

The coupling of autophagic machinery to EV secretion may also have evolved as a mechanism to expel unwanted or harmful material when the lysosome function is overwhelmed or impaired. Indeed, the enhanced secretion of autophagic cargo in response to PIKfyve inhibition may relate to critical functions of this kinase in late endosome trafficking and lysosomal fusion.^46^ This is consistent with the observation that EVs from apilimod treated cells are also highly enriched in proteins modified with ubiquitin, a covalent modification that typically earmarks proteins for lysosomal and/or proteasomal degradation. In addition, recent evidence suggests that Sirtuin‐1 mediated deacetylation of the V_1 _V_0_‐ATPase complex is critical for lysosomal acidification, and the loss of this SIRT1 expression in cancer cells contributes to EV secretion of ubiquitinated cargo that would typically be destined for degradation.^50^ Similar mechanisms may also contribute to the release of autophagic cargo in age‐related disease such as neurodegeneration, which are associated with a decline in lysosome function.^51^

Although the ESCRT machinery plays critical roles in many EV biogenesis pathways and is implicated in both classical autophagy and microautophagy,^18^, ^52^, ^53^ most ESCRT components appear dispensable for LDELS (Figure 2). This unexpected finding suggests that ESCRT components, or other mechanisms, function redundantly to execute LDELS, and genetic depletion of individual components may not be sufficient to impair this EV secretion pathway. Indeed, a targeted siRNA interrogating the role of ESCRTs in the release of CD63 and MHC class II suggests that components of this machinery may be significantly more redundant than initially envisioned,^54^ which one may expect given the critical functions of ESCRTs in numerous cellular processes distinct from EV secretion, including membrane repair and cellular abscission.^55^ On the other hand, our initial results suggest that LDELS utilizes an alternative ceramide‐dependent EV biogenesis mechanism that operate separately from ESCRTs.^49^ Indeed, one can speculate that this use of ESCRT‐independent pathways for EV cargo loading in LDELS may afford important biological redundancies. For example, in response to viral infection, the ESCRT component TSG101 is subject to inhibition through modification with the interferon inducible ubiquitin‐like protein ISG15.^56^ This modification targets TSG101 for autophagic degradation, suppresses exosome secretion, and may functionally inhibit viral exocytosis. In this context, LDELS would remain intact and hence contribute to ATG‐dependent antiviral processes such as immune cell activation and antigen presentation. Undoubtedly, determining whether and how the ESCRT machinery and lysosome acidification impacts LDELS and more broadly influences ATG‐dependent EV secretion remains an important focus of future research.

Although the LDELS pathway involves an exosome‐like secretory mechanism, it seems likely the autophagy machinery also contributes to the biogenesis other EV populations in addition to classical exosomes. Accumulating evidence supports that EVs are a heterogenous mixture of vesicles that originate from distinct subcellular membrane compartments in response to specific cues.^2^, ^57^, ^58^ Broadly, EVs can be divided into three classes on the basis of physical size: exosomes (30–100 nm), microvesicles (100–1000 nm), and apoptotic bodies (500–2000 nm). Whereas exosomes are proposed to form at MVEs through machinery that drives intraluminal budding from the limiting membrane of this organelle, microvesicles and apoptotic bodies are formed via outward budding and blebbing of the plasma membrane, respectively.^2^, ^57^, ^58^ Indeed, roles for autophagy components at the plasma membrane and early endocytic structures raise the possibility that this machinery may also regulate the release of microvesicles and apoptotic bodies under certain conditions.^59^, ^60^, ^61^ For example, LC3 targeting to the plasma membrane during Influenza A virus infection facilitates filamentous budding of the virus at the cell surface.^62^ Moreover, autophagy pathway components are required for immunogenic cell death and, in addition to facilitating extracellular release of ATP, may contribute to immune activation via packaging of cellular components into apoptotic blebs.^63^, ^64^

## 7 PUTATIVE ROLES FOR AUTOPHAGY‐DEPENDENT EV PATHWAYS IN PHYSIOLOGY AND DISEASE

Growing evidence supports important roles for EVs in normal physiology and in pathological processes associated with human disease, including immunity, neurodegeneration, and cancer. Importantly, similar to autophagy, EV secretion serves as a fundamental homeostasis mechanism that facilitates the elimination of excess or unwanted cellular material. Furthermore, the material released in EVs has diverse functions in extracellular matrix remodeling and cellular signaling to impact tissues through cell non‐autonomous mechanisms. While the physiological role of LDELS and other modes of ATG‐dependent EV secretion remain an important subject for further scrutiny, in this section, we postulate how these pathways may operate as important regulators of cell and tissue function.

Many viruses co‐opt EV biogenesis pathways for the assembly of infectious particles and to establish host permissiveness. This is also the case with the autophagy machinery. Although autophagy predominately functions as an antiviral mechanism, numerous viruses usurp components of the autophagy pathway, most notably the LC3 conjugation machinery, to facilitate viral exocytosis or egress. Picornaviruses including poliovirus and coxsackievirus B co‐localize with autophagosome‐like membranes within infected cells and are released in microvesicles that also contain lipidated LC3, a process termed autophagosome‐mediated exit without lysis (AWOL).^65^, ^66^ Importantly, the packaging of picornavirus virions, which lack an envelope, inside microvesicles serves to shield the virus against immune detection and facilitate transmission. Enveloped viruses such as Epstein–Barr virus, varicella‐zoster virus, and human cytomegalovirus have also been shown to exploit components of the autophagy machinery to facilitate membrane acquisition during egress.^67^, ^68^, ^69^ Influenza A virus encodes an ion‐channel protein, matrix protein 2 (M2), which targets LC3 to the plasma membrane to support filamentous budding and virion stability.^62^ Finally, hepatitis C virus and dengue virus subvert LC3 family proteins and conjugation components to facilitate exocytic release through MVEs^70^, ^71^; viral exocytic pathways that show considerable similarity to LDELS. These examples not only highlight roles for various ATGs in the control of viral exocytosis but provide strong evolutionary evidence corroborating the autophagy machinery as a core regulator of EV secretion.

The autophagy pathway is also critical for maintaining protein homeostasis (proteostasis), particularly within post‐mitotic cells that are not protected by the dilutive effects of cell division.^72^ In the brain, impaired autophagy in neurons promotes the accumulation of toxic protein aggregates or inclusions linked to neurodegenerative disorders.^72^ Although the autophagy pathway is predominately thought to target neuronal aggregates for degradation in the lysosome, evidence also implicates ATGs in the secretion of aggregation‐prone proteins. For example, Alzheimer's disease (AD) is characterized by the accumulation of Aβ aggregates. Studies have revealed that a significant proportion of Aβ is generated at autophagic structures that contain amyloid precursor protein (APP) and γ‐secretase, the enzyme that mediates APP cleavage to Aβ.^73^ Furthermore, genetic disruption of *Atg7* in mice constitutively expressing APP leads to reduced secretion of Aβ and extracellular plaque formation.^74^ In addition, Aβ can be released via exosomes and lysosomal exocytosis.^75^, ^76^, ^77^ Taken together, these data intimate that, in AD, the autophagy machinery may contribute to plaque formation through EV secretion and secretory autophagy pathways, as opposed to classical degradative mechanisms. Similar to AD, the autophagy machinery has also been implicated in EV secretion of α‐synuclein,^78^, ^79^ an aggregation‐prone protein implicated in Parkinson's disease and Lewy body dementia. Moreover, EV populations associated with α‐synuclein release and intercellular transfer are enriched for p62/SQSTM1 and lipidated LC3, and secretion of these autophagic proteins is dramatically increased in the cerebrospinal fluid of Parkinson's disease patients.^79^ Nevertheless, it remains completely unclear whether ATGs are genetically required for EV secretion of α‐synuclein either under basal conditions or in the context of disease. Finally, the mutant, polyglutamine‐expanded form of Huntingtin (mHtt) causal in Huntington's disease has been shown to suppress exosome secretion in astrocytes and deregulate intercellular communication in neuronal tissue.^80^ Recent evidence implicating mHtt in autophagy inhibition suggests that this disease protein may impair exosome secretion in Huntington's via deregulation of the autophagy machinery.^81^, ^82^ Overall, delineating whether and how the core autophagy machinery promotes and suppresses neurodegenerative disease through EV biogenesis and secretory pathways remain key questions for further study. Accordingly, the discovery of LDELS undoubtedly provides an important molecular foundation for rigorously scrutinizing how specific LC3‐positive EV populations contribute to neurodegenerative pathologies.

Finally, autophagy is recognized to have a dual role in cancer, suppressing tumor initiation through the clearance of damaged and potentially harmful proteins and organelles, and also promoting tumor progression by facilitating cancer cell survival and adaptation to stress.^83^ Similar to classical autophagy, autophagy‐related pathways, including LDELS and other secretory autophagy pathways, are likely to impact tumor initiation and progression via EV secretion. Interestingly, one important mechanism through which autophagy promotes tumor suppression is by facilitating cellular senescence, a durable form of proliferation arrest triggered by stress.^72^ Transition to senescence involves profound cellular changes, most notably the acquisition of secretory functions that drive the release of pro‐inflammatory molecules and EVs, collectively referred to as the senescence‐associated secretory phenotype (SASP).^84^, ^85^ Evidence supports that the autophagy pathway is highly active in senescent cells and required for the SASP.^30^, ^86^, ^87^ Thus, it is tempting to speculate that the autophagy machinery facilitates EV production during senescence to limit the proliferation of damaged or stressed cells that are at risk for malignant transformation. In addition, tumor cells frequently exploit the classical autophagy pathway to sustain cellular metabolism and mitigate damage during periods of stress. Autophagy is particularly important in *RAS*‐ and *BRAF*‐driven tumors, owning to the heightened metabolic demands linked to these oncogenic pathways.^88^, ^89^, ^90^ Nevertheless, the autophagy machinery is also implicated in the secretion of factors that drive the invasion of *HRAS*‐transformed cells,^29^ although it remains unclear whether LDELS or other ATG‐dependent EV secretion pathways contribute to this phenotype. *HRAS* overexpression in epithelial cells promotes the packaging of mesenchymal markers (e.g., vimentin and matrix metalloproteinases) in EVs, which can induce EMT in recipient cells.^91^ In addition, the autophagy machinery may facilitate EV secretion in other oncogenic backgrounds. The LC3‐conjugation components ATG5 and ATG16L are proposed to regulate the secretion of EVs in mouse 4T1 mammary epithelial cells that can promote metastasis to the lung.^48^ Nevertheless, recent studies demonstrate that genetic ablation of LC3 conjugation components, which should disrupt both classical autophagy as well as ATG‐dependent EV secretion, results in enhanced metastasis via numerous breast cancer cell‐types.^92^, ^93^ Hence, further work is needed to illuminate how the autophagy machinery controls degradation and secretion in cancer cells during metastatic progression; all of which will be critical for resolving the best avenues and for identifying targets, which may control both autophagy and autophagy‐related pathways, that can ultimately be leveraged for therapeutic benefit in malignant disease.

## 8 CONCLUDING REMARKS

The emerging role of the core autophagy machinery in secretory autophagy and EV secretion reinforces the complex functionality of ATGs in the elimination of cellular material and further implicates this pathway in the systemic regulation of homeostasis. Nevertheless, how these secretory pathways are regulated and what functions they serve in tissue and systemic homeostasis remain largely unknown. Certainly, much is left to discover about the roles that ATGs play in health and disease, and how this disposal machinery can be reconfigured to control cellular secretion and more specifically, the loading and biogenesis of EVs.

## CONFLICTS OF INTEREST

JD is the member of the Scientific Advisory Board of Vescor Therapeutics, LLC.
